# Living with Long Term Conditions from the Perspective of Family Caregivers. A Scoping Review and Narrative Synthesis

**DOI:** 10.3390/ijerph18147294

**Published:** 2021-07-08

**Authors:** Patricia Marín-Maicas, Silvia Corchón, Leire Ambrosio, Mari Carmen Portillo

**Affiliations:** 1 Faculty of Health, Valencian International University, 46002 Valencia, Spain; pmarinm@universidadviu.com; 2 Faculty of Nursing and Chiropody, University of Valencia, 46010 Valencia, Spain; 3 NIHR ARC Wessex, School of Health Sciences, University of Southampton, Southampton SO17 1BJ, UK; L.Ambrosio-Gutierrez@soton.ac.uk (L.A.); M.C.Portillo-Vega@soton.ac.uk (M.C.P.)

**Keywords:** living with long term conditions, experience, family caregivers, person-centred care

## Abstract

(1) Background: When living with one or more long term conditions (LTCs), both the patient and the family experience the impact of the condition at different levels. The family’s needs and perceptions should be considered in the process of caring for people with LTCs. The aim of this review is to understand “the process of living with LTCs” from a family perspective. (2) Methods: A scoping review and narrative synthesis were conducted using a systematic methodology in MEDLINE, CINAHL, Web of Science and PsycINFO, in English and Spanish, including evidence from 2018. (3) Results: A total of 28 articles were included in the review. Acceptance, coping, self-management, integration, and adjustment were key attributes in the process of living with LTCs from the perspective of family caregivers that interrelated in a dynamic way through different mechanisms: being aware of the changing situation, personal networks, information and education, personal conditions, attitude to life and communication. (4) Conclusions: The five attributes that comprise living with LTCs from the perspective of the family caregiver are closely connected of to those of patients living with LTCs; however, self-management and integration have a different meaning and application.

## 1. Introduction

Population aging is the most important demographic phenomenon of recent decades, producing changes in social and epidemiological patterns [1]. The structural changes that the world’s population has undergone have led to the growth of long-term conditions (LTCs) [2]. The vision of LTCs has evolved over the years, as a result of the development of different countries and the social and health transformation. An LTC is now defined as a process of long duration and slow progression that requires continuous and lasting care and treatment [3]. Currently, LTCs contribute approximately 60% of the total 56.5 million reported deaths worldwide and approximately 46% of the global burden of disease and cost to health systems [2,4,5]. With a rapidly aging global population, the demands on health services to address disability outcomes, which increase with age, will require policy makers to anticipate these changes [4]. As disability becomes an increasingly important component of the disease burden and a larger component of healthcare expenditure, new strategies are needed to improve care for people with LTCs and their family caregivers [1,4,5,6]. Patients and family members live with one or more LTCs for many years because living with an LTC is not only an individual concern, but also a family affair [7,8,9,10]. 

Family caregivers’ perspective need to be considered when planning policy and health and social care guidelines because it is estimated that up to 80% of all long term care in Europe is provided by informal caregivers [9], and these figures follow similar trends worldwide [11]. A family caregiver is considered *“a non-professional person who provides primary assistance with activities of daily living, either in part or in whole, towards a dependent person in his/her immediate circle”* ([12], p. 2). The family is the environment where the living experience takes place and is managed [13]. Current evidence argues that there is a need for comprehensive policies that capture the family caregiver of people with LTCs, directing measurement tools and interventions to this population [3,5,8,10,14]. At present, caring for people with LTCs constitutes one of the most important challenges facing health and social care systems around the world [3,15,16]. In this sense, health and social care professionals should adopt a multidimensional approach to care, addressing the complexity of the person as a biopsychosocial and spiritual being [17,18]. To include care for the family caregiver of people with LTCs is one of the strategic objectives to assist in the management of chronicity and multimorbidity [3,8,9].

Previous conceptual work [19] on living with an LTC from the patient’s perspective has been used as a reference for this review, considering this experience of LTCs a complex, cyclical, dynamic, constantly changing process that affects people in all the spheres of their life [18]. Furthermore, Ambrosio et al. [19] identified five attributes, namely acceptance; coping; self-management; integration and adjustment that were key aspects of the process. From the patient perspective, *Acceptance* involves being aware of the condition and becoming prepared to face the changes that it will generate. *Coping* is, refers to how the person develops and implements strategies to deal with the chronic process. *Self-management* requires that the patient has knowledge about the disease, can follow a treatment plan participates actively in decision-making and knows how to solve the adversities that the disease generates. *Integration,* in addition to the above considers the disease part of the person’s life, providing a new sense of normalcy in life. *Adjustment* is considered the last attribute of Living with LTCs process, because in addition to all the previous ones, it leads to a process of transformation in the person, materializing a change of life [19]. The final goal is achieving a ‘positive living’ with LTCs, according to their situation and/or context [19] promoting quality of life and well-being. Despite the extensive literature published in the last years regarding family caregivers, we have not found an article that addresses a conceptual understanding of the process and these attributes from the perspective of the family caregiver. Therefore, this review will attempt to answer the review question: “What is the perspective of family caregivers on living with LTCs?” with the aim of finding out how living with LTCs is understood from the perspective of the family caregiver and what factors could influence this process.

## 2. Materials and Methods

A scoping review with systematic methodology was developed [20,21]. This review was assessed through following the Preferred Reporting Items for Systematic Reviews and Meta-analyses extension for Scoping reviews criteria (PRISMA-ScR) [22]. 

To identify the relevant literature, a search strategy was designed. The databases searched included Medline (PubMed), PsycINFO, CINAHL (Ebsco), Cochrane, and Web of Science. Search strategies were translated using each database platform’s command language, controlled vocabulary, and appropriate search fields. 

### 2.1. Identify the Research Question and Inclusion and Exclusion Criteria

Starting from the review question: What is the perspective of family caregivers on living with LTCs? We follow the mnemonic PCC (population, concept and context) to identify the focus and context of the review [20,21]:

- POPULATION: Family with an adult relative with LTCs.
- CONCEPT: Living with LTCs from family perspective.
- CONTEXT: Quantitative, qualitative, mix methods studies, political documents that totally or partially address the meaning and the experience of living with LTCs from the family caregiver perspective.

Consequently, MeSH terms and text words were used for the concept of family caregivers and the concept living with LTCs. The search strategy was constructed using the boolean operators AND and OR as outlined below in Table 1.

Inclusion and exclusion criteria: We included different types of studies to map the evidence about the perspective of family caregivers living with LTCs, and excluded studies that only focused on living with from the patient’s perspective, or focused on paid or formal caregivers or on informal caregivers with no link to the person with LTCs. Moreover, although adulthood has been used as a limit, numerous results from articles referencing family caregivers of younger patients (parents) appeared in the search. Therefore, this was also established as an exclusion criterion (see Table 2). 

### 2.2. Procedure

The studies were selected through a two-step process. First, each of the titles and abstracts were reviewed based on the criteria of relevance to the study topic. Then, the full text of the selected citations was assessed in detail using the inclusion criteria, and PM-M validated the selection of the articles with two reviewers (S.C., L.A.). Any disagreements that arose between the reviewers were solved through discussion with the member of the group who was the expert in LTCs (M.C.P.). Following the search, all identified records were collated and uploaded into Refworks, and duplicates were removed. During the process, articles found by manual search and using the snowballing technique were incorporated. 

Following the PRISMA-ScR recommendation [22], quality appraisal was assessed using the Joana Brigs Institute critical appraisal tools [20]. Validation of the results took place following the same procedures as indicated above for the selection of papers.

The data extracted included specific details about the population, setting, methods and key findings relevant to the review, which was conducted using a tool based on the Cochrane tool [25]. The most relevant results are shown in results section.

### 2.3. Analyses

A narrative synthesis was performed to synthesize the findings of the included studies. Taking the existing conceptual framework on living with LTCs from the patient’s perspective as a reference [19], this review focused on the meaning of living with LTCs from the family caregiver’s perspective. Deductive and inductive thematic analysis [26] took place in order to synthesize the included papers as described below. Braun, Clarke and Weate state that: “in practice, most thematic analyses include both semantic and latent, and inductive and deductive elements” [27] (p. 4). Combining two approaches allowed the development of patterns from the unknown parts that may be left out of the prediction codes of deductive reasoning, providing a complete picture of the process [28].

As a starting point, a deductive thematic analysis [26] of the results and discussion text of the included papers was undertaken to identify what aspects of the process of living with LTCs from the patient’s perspective [19] applied to family caregivers, looking for similarities and anomalies [28]. Ambrosio^]^) et al.’s [19] previous work on attributes that define the process of living with LTCs for adults patients was used in this phase of the analyses as a reference framework leading to proposing, one theme and five subthemes were created.

Following this, an inductive thematic analysis of the reviewed papers was also developed because it was paramount to capture further constructs and aspects that were unique for family for family caregivers when living with a person with LTCs. Applying the 6 steps suggested by Braun and Clarke [27], the inductive approach in this analysis focused on patterns from the “facts” or raw data of the papers that led to further understanding of the phenomenon of living with LTCs and complemented the existing framework. A total of one additional theme and six subthemes emerged from this phase of the analysis Validation of the results took place following the same procedures as indicated above for the selection of papers with the team.

In total, after both phases of the analysis were completed two themes and 11 subthemes emerged, as illustrated in Table 3.

## 3. Results

**Figure 1:**
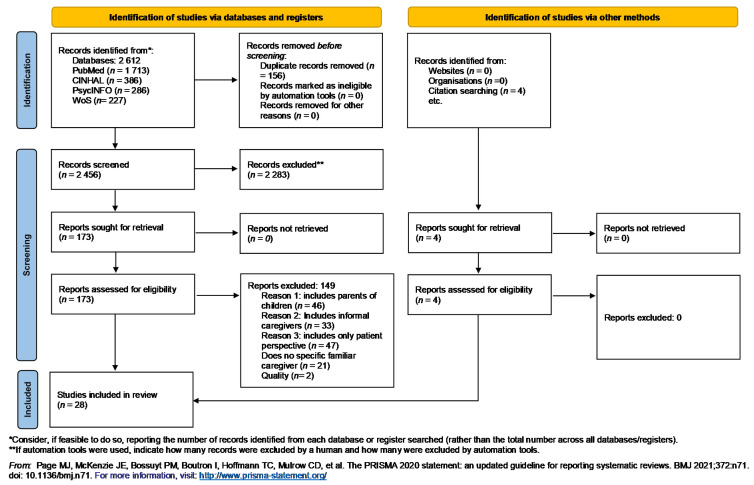
PRISMA 2020 flow diagram [29].

For this review, 2612 articles were retrieved. After eliminating duplicates, selecting by relevance, applying the inclusion and exclusion criteria and assessing the quality, 28 articles were finally included in the review (See Figure 1). 

Regarding the critical appraisal of the individual sources of evidence, two of the selected articles did not meet the appropriate quality criteria and were excluded (one for not including ethical approval of the work [30] and one for not adequately describing the data collection methods [31]. Included articles scored YES on most items of the Joana Brigs Institute tools, including some studies with at most two negative responses. We found some weaknesses in the qualitative studies regarding the presence of the research question in the reviewed articles, as most of them included objectives but no research question. As for the quantitative articles included in this review, all of them showed high compliance with the Joana Brigs Institute tool items [20]. Finally, of the included reviews, the least frequently found item was the method for minimizing errors during data extraction and methods for combining studies. The papers included were 19 primary studies (11 qualitative studies, six quantitative studies and two mixed-methods studies) and nine reviews (four systematic reviews, one meta-analysis, one metasynthesis, one scoping review, and two integrative reviews). Of the 28 studies, 22 of them included sociodemographic data on participants. A total of 28,226 family caregivers were included, of whom approximately 71% were women and 29% men. Thirteen of the 28 articles reported on the mean age of the participants, resulting in an overall mean of about 55.35 years. Of the 17 articles indicating the degree of relationship between the patient and the family caregiver, 89.5% were spouses, 3% were sons and daughters, 0.8% were siblings and 6.6% had another relationship (including parents, grandparents, grandchildren, daughters-in-law, sons-in-law or best friend).. Fourteen studies included the diagnosis of the patient being cared for, the most representative being cancer (77%; 7568/10,006), followed by cardiovascular disease (8.5%; 863/10,006), mental disease (1.71%; 186/10,006) and COPD (1.15%; 113/10,006). Details are presented in Table 4.

The results of the review are presenting following a narrative synthesis, with two themes and eleven subthemes emerging related to living with LTCs from the perspective of the family caregiver. Table 5 includes some original quotes (Q) and text segments (T) from the articles to illustrate the themes and subthemes and increase the rigor and transparency of the analytic process. 

### 3.1. Attributes of Living with LTCs from a Family Perspective:

In this theme, an attribute is understood as a characteristic or inherent quality of something. In this context, an attribute is considered as a characteristic of the concept of living with LTCs. Five attributes comprise the concept of living with LTCs.

*Acceptance* as an attribute was found in nine of the 29 articles reviewed (Table 3). Accepting occurs when family caregivers understand the cause of the problem, embracing the reality of life and all experiences [33] (Table 5, T_1_, Q_1_). Different relevant moments that acceptance gains importance have been identified in the review papers. It involves assuming and normalizing the role of caregiver [38] and being prepared to adapt to the changes that occur on a daily basis [35,36] (Table 5, T_2_, T_3_ and T_4_). According to Moral-Fernandez’s work, acceptance would be essential at the initial stages of care when people take on the role of family caregiver [38]. The transition process that a family caregiver goes through is marked by the changes that occur [32,38,51]. Accepting the new role makes it possible to achieve a situation of normality when living with LTCs [34,38]. Moreover, Dekawaty et al. [33] relate acceptance to an individual and final stage of adaptation [33]. Roberts and Struckmeyer [56] describe acceptance as the ability to step back from a caregiving situation to assess the entirety of the situation (Table 5, T_7_). In their work about caregiver respite, they note how the pathway to acceptance can become a critical factor in the development of resilience for family caregivers [56]. Family caregivers generally stand between acceptance and resistance to the care situation [36,38] (Table 5, T_5_ and Q_2_). Roberts and Struckmeyer [56] also highlighted this point describing how caregivers reach a point where they face the duality of having to fight their situation or accepting it to move on. Bertschi et al. [54] show how the lack of acceptance hindered coping. 

Coping is an attribute found in 10 of the 29 articles reviewed (Table 3). Coping in family caregivers involves implementing strategies to minimize the negative effects of caregiving that allow them to overcome the problems that arise during caring routines [38]. Once family caregivers have assumed the role, they move on to a new stage in which family caregivers implement strategies to minimize the negative effects of caregiving [38] wich allow them to cope with the social, emotional, physical or other problems that arise during care. (Table 5, T_6_, T_10_ and Q_3_). Coping is dynamic, oriented to solve the problems that arise from changes in the family member’s health and depends on both own and external resources [33,36,38] (Table 5, T_8_–T_10_ and Q_4_). In particular, Dekawaty et al. [33] highlight that coping *“involves aspects such as the nature of the stressful or the stimulus itself, personal characteristics and external resources such as the support received” (*p.8). Different coping strategies have been identified in the reviewed papers. Cognitive coping strategies from the family caregiver perspective include praying, valuing life or having hope [33,34,40] (Table 5, T_16_). Behavioral strategies like seeking help [32] and problem solving [38] (Table 5, T_11_). Moreover, Roberts and Struckmeyer [56] underline that self-efficacy, hopefulness and stress resistance as necessary components of coping capabilities, a complex construct which is often referred to as resilience. Concretely, caregiver’s resilience refers to how caregivers use effective coping strategies, transforming the burden of caregiving into strengths [56]. Dialogue and communication are presented as a fundamental element for the correct coping of the living, with processes both between couples [36,50] and between the different members of the family [7,37,40,43]. These was also identified by Helgeson et al. (2018), Dekawaty et al. [33], Bertschi et al. [54], Gibbons et al., [55] and Riffin et al. [52] who describe how dyadic or communal coping may favor adaptation to LTCs [33,50,52,54,55].

The *self-management* attribute emerged in 11 of the 29 articles reviewed (Table 3). Self-management for family caregivers involves balancing care of the family member and themselves [33,50,51]. Nevertheless, maintaining balance while caring for a chronically ill person could be challenging [51] (Table 5, T_21_, T_26_, T_31_, Q_5_). The studies reviewed show that family caregivers have limited time to maintain their physical health [36] and often tend to put the interests of their sick relative before their own, leading to a reduction in their quality of life [32,36,38,40] including neglecting health self-examinations [33] (Table 5, T_26_, T_28_, T_31_). Although the uncertainly of the process may unbalance family caregivers’ self-management capacity [36,42] (Table 5, T_24_, T_26_, T_29_), unlike family support [32,39] (Table 5, T_25_, T_27_, T_30_). Kusi et al., Gillis et al. and McGlinton et al. [40,41,42] related that self-management refers to the role that family caregivers adopt in the management of the patient’s symptoms and illness (Table 5, T_17_, T_18_, T_19_, T_20_). Family caregivers are often seen filling system gaps to provide care, monitor or encourage the patient to achieve goals [41]. In this sense, Kusi et al. [40] found how acquiring knowledge about the pathology may help family caregivers with their caregiving role (Table 5, T_23_).

*Integration* was as an attribute found in six of the 29 articles reviewed (Table 3). From the point of view of family caregivers, integration involves creating the adequate environment to self-manage the illness [39,44] (Table 5, T_37_, T_38_, T_39_, T_40_). The processes and perceptions of living with LTCs influence the family as a functioning system [44]. Families, and thus family caregivers, can find benefits in the context of the LTC experience over time, and this is an ongoing process [7,44] (Table 5, T_35_). Eriksson et al. [36] state that integration from family perspective involves balancing daily chores, maintaining the support of the social network and adapting to constant changes and uncertain future (Table 5. T_32_–T_34_, and Q_6_). Implementing strategies to minimize the effects of care, reorganizing relationships at the family and social level, learning skills to be self-sufficient in caring and solving problems that arise daily mark a new situation of normalcy for the family caregiver [38]. The personal growth and satisfaction that the task of caring brings, is an aid to value the things of the day to day [35,38,39]. (Table 5, T_36_, T_37_, T_41_, Q_2_, and Q_7_).

Finally, the attribute of *adjustment* was reported in eight of the 29 studies reviewed (Table 3). Adjustment to illness for family carers involves maintaining balance in family life and in roles when challenges arise [32] and involves accepting the condition and striving to live together [33] (Table 5, T_42_, Q_8_). Adjusting to a new normal involves tackling and disabling health problems on a daily basis [54] (Table 5, T_44_). Psychosocial adjustment to the disease is a person-centered process rather than a disease-related process, showing how social functioning, mental health and vitality are fundamental to achieving adequate adjustment [48] (Table 5, T_43_). Family functioning and resilience are psychosocial factors that are associated with adjustment to illness [47,48,56], and Whitehead et al. [39] found a predictive relationship between family adaptability and family caregiver depression (Table 5, T_44_, T_45_, and T_47_). Arested et al. and Zhang [7,47] point out that the family adjustment is possible when good relationships between family members are present and adequate family functioning is achieved. Communication and adaptability to changes are two key elements in adjustment [37,47].

### 3.2. Mechanisms of Living with LTCs from the Family Perspective

In this theme, a mechanism refers to enablers and barriers to living with LTCs from a family caregiver perspective and how they interact. Six mechanisms emerged from the data analysis of the articles:

*Awareness of the situation of change created by LTCs*: As reported in 10 articles [7,32,33,34,35,38,39,40,49,51], family caregivers are directly involved in the lives of their relatives [32], especially spouses, where care is generally intensive, and many have no choice when it comes to assuming the role of caregivers [49]. The onset of the disease can sometimes come as a shock for family caregivers, precipitating a change in their lives (Table 5, T_49_–T_52_ and Q_10_). This is projected as a transition process marked by the changes that take place [32,38,51] and uncertainty [32,35,38,39], fear [40] and negative and positive emotions are present [38]. The motivation to caring is an element that can condition acceptance, finding that the sense of responsibility for caring for relatives is the basis of this commitment [34] (Table 5, T_46_ and Q_9_). Sociocultural characteristics and the environment can influence the involvement and motivation of family caregivers [7,32,33,34,39,40,49], since accepting the new role makes it possible to achieve a situation of normality living with LTCs [34,38]. 

*Personal networks*: In 13 articles, several authors have argued the importance of personal networks in the transition process of living with LTCs from the family caregiver perspective [7,11,32,33,35,36,37,40,42,45,47,55,56]. The networks identified in the selected studies are family friends and professional support and other social contacts (Table 5, T_57_–T_61_). Roberts and Strukmeyer [56] additionally identify support groups and respite care programmes (vouchers) as a key community resources for family caregivers (Table 5, Q_11_ and Q_12_). The deterioration of social relationships leads to isolation, which increases the associated burden of their caregiving role [36,37,40,56] (Table 5, T_62_). Helping supporting the family caregiver to manage their social life seem key facilitators in healthy lifestyles and living with LTCs. [51]

*Information and education about the LTCs:* Seven articles have highlighted that information is a key aspect for family caregivers [11,32,36,38,40,41,42]. The lack of information and preparation for the situation, especially in the initial phase of caregiving, is especially relevant [32,38,42] (Table 5 T_58_, T_63_). Knowledge about the disease is essential for acceptance of the process and helps to reduce the burden on family caregivers [40] (Table 5, T_64_, T_65_). Due to the lack of information, family members can find that they sometimes have to assume an advocacy role, needing to be involved in acquiring medical and service information and in decision making, increasing their feeling of burden coordinating care and services for the patient [11,41,42]. 

Personal conditions: In nine articles, [7,11,32,33,34,38,40,42,49], several authors have described how previous experiences, socioeconomic conditions, personal beliefs and the sociocultural meaning of care can influence how family carers live with LTCs (Table 5, T_43_, T_66_, T_67_, T_68_, T_69_, T_70_, and T_71_). Sociocultural characteristics and environmental factors may influence the involvement and motivation of family caregivers (i.e., the cultural meaning of care, the sense of responsibility, love for those with LTCs or cultural norms) [11,33,34,40]. 

Attitude to life: Seven studies have underlined that maintaining an optimistic attitude can enable the process of living together [11,32,33,36,38,39,41] Dekawaty et al. [33] describe a positive attitude as a characteristic of acceptance (Table 5, T_75_). Particularly, maintaining a positive approach to the LTCs and not focusing on the problem is related to the positive emotions involved in caregiving such as love [32], marital satisfaction [41] or personal growth [36] and helps one appreciate the value of the everyday [38]. Whitehead et al. [39] describe that family involvement helps to maintain a positive attitude (Table 5, T_72_) and this fact bring the opportunity to develop a closer relationship with their family member, reciprocate the provision of care or help their family member to stay at home, avoiding hospital and care home admission [11] (Table 5, T_73_, T_74_). 

Communication: The importance of communication for family caregivers has been found in 14 articles [7,11,35,36,37,39,40,42,43,44,45,50,51,52,54]. From the family communication perspective, the results shows how dialogue and communication are perceived as a fundamental element to embrace appropriate coping skills in couples [36,50] and among different members of the family [7,37,40,43,52]. Difficulties in communication derived from the LTCs pose an added difficulty for the family caregiver [54]. Riffin et al. [52] found how disagreement regarding LTCs between the family caregiver and patient can negatively affect their relationship, leading to poor outcomes in the adjustment process. Qin et al. [45] showed how divergent views on the management of LTCs can converge into a major source of stress. According to Bertschi et al.’s [54] in cases where family caregivers cannot accept the situation and embrace the new role, deterioration in communication or limited mutual support could jeopardize acceptance and consequently, coping skills. Facilitating communication between patients and spouses influences how couples face chronic misunderstanding about illness and symptom management and can involve emotional release, foster support and help build intimacy [43] (Table 5, Q_13_). Additionally, the presence of uncertainty among family members in relation to living with LTCs has been related to poor the effectiveness in communication among family members [39] (Table 5, T_77_–T_79_). 

From another perspective, some studies [7,11,42] identify the how valuable communication with healthcare providers is for family caregivers (Table 5, T_80_). The patient is often expected to navigate autonomously through the system, disregarding language and understanding barriers they may have [11]. Family caregivers have frequent encounters with healthcare [7] and express that, sometimes, there is poor communication with healthcare professionals which leads caregivers to feel they need to actively intervene [7,11,42]. Families (and people with LTCs) can experience lack of information and communication with healthcare professionals [42], and this is even confirmed by healthcare professionals [7]. Helping with communication skills [37,47] seem key facilitators in living with LTCs.

## 4. Discussion

In response to the review questions, we could state that the perspective of family caregivers on living with LTCs is closely connected to the patient’s perspective. The five attributes previously proposed to patients living with LTCs have been also found when looking at evidence on family caregivers. Furthermore, some further understanding of the Living with LTCs process has been developed from the family caregiver`s angle.

Acceptance in the family caregiver consists of assuming and normalizing the caregiving role, which is described as an essential element for achieving an adequate adaptation [33,38]. Although the present study has included results from research conducted in different cultures and countries, this may be a limiting factor in the results found. Concretely, motivation can condition acceptance [34], but both the involvement and motivation in family caregiving can be culturally diverse across countries and cultures [7,32,34,39]. In the current globalised world, may it necessary to include evidence that represents the reality from a multicultural perspective. This mapping of evidence describes common elements of care from the point of view of different cultures in order to provide health and social professionals with a broad, comprehensive and diverse view of the phenomenon living with LTCs from family perspective.

Additionally, once family caregivers have assumed the role, they implement strategies to minimize the negative effects of caregiving that enable them to cope with problems that arise during caregiving [38], therefore, acceptance is the first attribute necessary to achieve positive living with LTCs. This finding is congruent with previous studies about living with LTCs from family perspective [58] and also from the patient [19], so assessing the degree of acceptance of LTCs in both patients and family members may be essential in care planning.

Both behavioral and cognitive coping strategies found in relation to the family caregiver have been found relevant to Lazarus and Folkman’s coping theory [59]. This fact indicates that family caregivers have multiple options to develop coping strategies, but the response is individual and different for each person. Therefore, this fact health should consider by health and social care professionals when assessing and planning care. Furthermore, communication emerges as the key element for a correct coping process [7,36,37,40,43,50]. A lack of communication can be an added difficulty in the process of living with LTCs between the person with LTC and his or her family, leading to a deterioration in the relationship, and this finding is consistent with the previous work of Checton et al. [60] and Arested et al. [13]. On the other hand, when an LTC is defined as a single person’s problem, interactions between family members may be imbalanced [50]. In this sense, community and dyadic coping is described as an opportunity to improve coping in LTCs [33,50,51,54]. This finding is in line with the latest recommendations included in several current Clinical Practice Guideliness and policy documents in which it is assumed that LTCs are a problem that affects not only the patient but also the whole family, and therefore the assessment and coping strategies should be take place conjunctly [2,4,8,61]. Acceptance can trigger for coping [38] and both are needed for achieving an appropriate balance between resources and self-management demands. Therefore, acceptance is indirectly related to the other attributes. Self-management from the patient’s perspective has a different meaning than from that of the family caregiver. From the patient’s perspective, self-management includes coping with the LTC, also implementing strategies, having knowledge of the LTC, actively participating in the decisions and solving the problems that arise [19]. Self-management from family caregivers’ self-manage their health [36,39,51] but also their role dealing the patients ‘symptoms and illness [40,41,42]. In order to remain healthy the family caregiver needs to balance care for the ill family member and also for oneself [36,50,51]. In this review, preserving social and family relationships [7,33,36,39] brought meaning and satisfaction to the life of family caregivers [36]. This has previously highlighted in the Theory of Salutogenesis described by Antonovsky [62]. Therefore, supporting the family caregiver to manage social life could potentially facilitate health and promote a more positive living with LTCs [51]. On the other hand, family members often develop aspects of care, substituting or complementing professional services to encourage the patient to achieve goals [41]. Consequently, many patients rely directly on care provided by the family caregiver and acquiring knowledge about the pathology and developing the necessary skills to perform the task, may help family caregivers cope effectively with their caregiving role [38,40]. Thus, for the family caregiver, self-management should include their care as the management of their family member’s LTCs. This finding marks a difference with the self-management concept described from the patient’s point of view that future research should explore and work to integrate this new meaning into actions aimed at empowering self-management in family caregivers.

Acceptance and coping are necessary to achieve a positive living [34,38], and family collaboration enhances self-management, helping to build an environment of normality (integration), contextualization of the LTC and adaptation [7,39,44]. The family caregiver is part of a family unit, and therefore the results found from the family functioning perspective may be transferable as family functioning influences all family members [7,29,36,41]. In this sense, the integration attribute in living with LTCs for family members is closely related to the concept of family functioning. Positive family functioning and increased support among family members may contribute to a lower perception of caregiver burden and a higher perception of well-being. [45] Integration is described as an essential element in achieving adequate adaptation [30,35]. This review findings regarding the Adjustment to the illness attributes are congruent with Patterson and Garwick’s work [63] which indicated that maintaining balance in family life and roles when challenges arise, accepting the condition and being able to live normally with the new situation by coping with daily problems [29,30,51]. To achieve adequate psychosocial adjustment, social focus, mental health and vitality are also necessary [45]. Helping with communication skills [34,44] and helping the family caregiver to manage social life seems to be a way to facilitate health and foster positive living with LTCs through care plans and thus enable adjustment [48]. 

**Figure 2:**
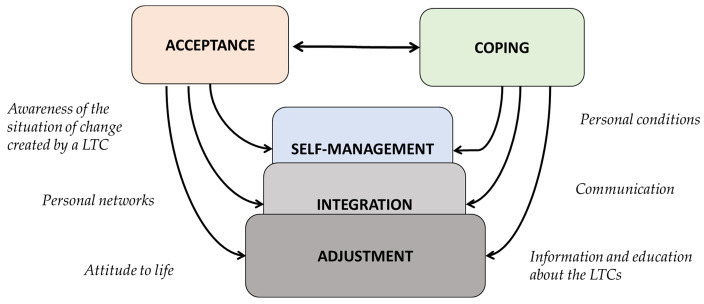
Interrelationships between the attributes of living with LTCs from the perspective of the family caregiver and mechanisms involved.

As described above, the attributes involved in the process of living with LTCs from the family caregiver perspective are interrelated and dynamic. The results obtained do not show a linear relationship between the attributes from the family caregiver perspective, which makes the process complex in a similar manner as to the patients [19,64]. This complexity extrapolates with multiple mechanisms that can trigger a positive or negative response, and also need to be contemplated in care assessments, plans and guidelines. Following the findings described above on how attributes are related and regarding how mechanisms can influence them, the non-lineal process has been depicted in Figure 2.

Gender has implications for health across the course of every person’s life [65]. Women are the main caregivers in the family, providing the majority of care [66,67] (i.e., hours of dedication, loss of working). This fact, implying that most of the caregiver studies are conducted on women and perhaps this fact may produce a gender bias on the perceptions. For example, women tend to use coping strategies that are aimed at changing their emotional responses to a stressful situation, whereas men use more problem-focused or instrumental methods of handling stressful experiences [68]. As the authors do not analyse gender perspective in the included articles, this factor should be enlarging in relation to how caregivers live with chronic illness.

The impact of illness is variable, and so is the process of living together from the perspective of family members [32]. The constant worry about the sick family member proves to be exhausting for the relatives [29] and often appears as stress reflected in physical and emotional problems [32,33,34,37,43]. The continuous burden can cause physical, psychological and economic problems for the family caregiver if the necessary mechanisms are not in place [29,30,35,37,49,50]. Encouraging positive living with LTCs in family caregivers can help to avoid the burden of care, so efforts designing of health and social policies in the management of LTCs should go in this direction by including interventions in clinical practice aimed at both the patient and the family caregiver.

This new conceptual understanding of the process of living with LTCs from the family caregivers ´perspective supports existing health and social care policy [2,61,69], by identifying key outcomes that matter to family caregivers, illustrating the process of living with LTCs from their perspective an those elements and mechanisms that need to be prioritized when distributing resources in health systems, personalizing care plans for families and providing guidance on how to empower family carers to take further control of their role and impact in decision making. Although this review focused on family carers of people with LTCs, our findings are also applicable to dementia and fragility management [10].

## 5. Limitations

One limitation of this review is the year of publication range of the articles included in the review because only the last three years have been included. This decision was made to ensure this review synthesized the most current evidence on the topic of the study since family caregivers have been extensively studied in the last decades. Furthermore, different terms used by the authors to refer to similar concepts may also have complicated the interpretation of the data; we overcame this situation by sharing the findings in group meetings and seeking validation. Finally, the limits of language used in this article may have left out some articles available in different languages.

## 6. Conclusions

The five attributes that comprise living with LTCs from the perspective of the family caregiver are related to those of patients living with LTC. However, self-management and integration have a different meaning and application. This new knowledge on the attributes and mechanisms that aid in living with LTCs from the family caregiver’s perspective is essential in sustainability and transformation plans in health and social care services to develop more meaningful programmes for personalized care strategies, which could have a positive impact on family functioning and health and self-collective efficacy when managing LTCs. 

## 7. Relevance for Clinical Practice

This paper highlights the importance of a holistic approach to LTCs for the patient and their family and describes the different mechanisms that could help the family caregiver. Including the family caregiver as part of plans and guidelines may help families to achieve adaptation to LTCs while maintaining family functioning and health. This innovates understanding of how family lives with LTCs needs to be integrated in existing clinical practice assessments, templates and personalized care programmes. New assessment tools may also need to be developed in line with these new findings to enhance the referral process not only for patients but also for family carers to specialized care or parallel resources in the community. More concretely, considering these elements of the process of living with LTCs and acknowledging families perceptions of the process could lead to more timely referrals, improved systems of support and more comprehensive understanding of the support needs and networks patients with LTCs and their families have in the community, leading to more targeted interventions and fewer admissions and direct/ indirect costs related to informal caring, multimorbidity management and carer burden.

## Figures and Tables

**Table 1:** Detailed search strategy.

Search	Query
#1	(((“FAMILY CARER*”)) OR (“SPOUSE”[All Fields])) OR (“FAMILY CAREGIV*” OR COUPLE OR RELATIVE* OR “INFORMAL CARE*“OR CAREGIVER* [MeSH Terms])))
#2	((“LONG* TERM CONDITION*” OR “CHRONIC DISEAS*” OR MULTIMORBIDITY OR “CHRONIC CONDITION*” OR “LONG* TERM ILLNESS*” OR “CHRONIC ILLNESS*”))
#3	((NEED* OR COP* OR ADJUST* OR “LIV* WITH” OR EXPERIENCE* OR COEXISTENCE OR ACCEPT* OR ADAPT* OR INTEGRAT* OR PERCEPTION* OR PERSPECTIVE*))
#4	#1 AND #2 AND #3

Note: We used truncated terms (e.g., diseas*) to include all possible endings. Limits: Language limits were applied to capture articles in English and Spanish. To obtain the most current evidence on the topic of study, articles published in the last three years were reviewed by applying filter articles published from 2018 to the present. This year range was applied in a attempt to narrow our search to more recent years while looking for data saturation during the analysis of the included papers [23]. The final search was completed in December 2020 with a subsequent update to March 2021. An adult age filter was applied (age +19) because we started from a theoretical framework developed in the adult [19]. Moreover, there may be variations from the perspective of family caregivers of children with LTCs due to the legal implications, bonding and responsibility [24].

**Table 2:** Inclusion and exclusion criteria.

Inclusion	Exclusion
Quantitative, qualitative, mixed method studies, statutory documents totally or partially addressing the meaning and the experience of living with LTCs from the family caregiver perspective.	Studies that only show the point of view or the experience from person with LTCs.
	Studies that include experiences of family caregivers of younger patients (parents).
	Studies that include informal caregivers or remunerated caregivers.
	Studies that include family caregivers not living together. Studies containing more than two negative responses after assessing methodological quality with the JBI tool, or no ethical approval

**Table 3:** Themes and subthemes emerged.

Theme	Subtheme
1. Attributes	1.1 Acceptance 1.2 Coping 1.3 Self-management 1.4 Integration 1.5 Adjustment
2. Mechanisms	2.1 Awareness of the situation of change created by a LTC. 2.2 Personal networks. 2.3 Information and education about the LTC. 2.4 Personal conditions. 2.5 Attitude to life. 2.6 Communication.

**Table 4:** Characteristics of included studies.

Reference	Country	Sample	Type of Study: Study Design. Collection Methods.	Analysis Method Used	Attributes Found	Findings
García-San Juan et al., 2019 [32]	Europe	Adults, non-remunerated caregiver, relative caregivers living with those affected by Crohn’s disease. *n*= 11	Qualitative. Individual interview, snowballing. Maximum variation sampling	Thematic analyses.	Acceptance, self-management, integration.	It is relevant to know how family careers experience the process, showing a capacity to adapt to the uncertainty of the course of the disease. They find that the intensity of care is a risk factor (hours/week).
Dekawaty et al., 2019 [33]	Asia	Family caregivers living with Parkinson’s disease patients. *n* = 5	Qualitative. Individual interview. Purposive sampling	Thematic analyses.	Acceptance, coping, self-management, adjustment.	It addresses the meaning of caring for a Parkinson’s disease patient: coping, perceived stressors, family and social support and spiritual and cultural significance.
Salehi-Tali et al., 2018 [34]	Asia	Family caregivers living with hemodialysis patients. *n* = 16	Qualitative. Individual interview. Purposive sampling	Thematic analyses.	Acceptance, coping.	Addressing spiritual strategies, cultural beliefs may be related to the conception of pain and suffering. It concludes that the outlook of family caregivers is based on innate affection and love for the patient, representing aspects inherent in their beliefs.
Strang et al., 2018 [35]	Europe	Personal experience from caring for a COPD patient. *n* = 35	Qualitative. Individual interview and focus groups. Maximum variation sampling.	Thematic analyses.	Adjustment.	When the family has adapted, signs of happiness may appear.
Eriksson et al., 2019 [36]	Europe	Relatives living with patient with chronic disease. *n* = 16	Qualitative. Individual interview. Purposive sampling.	Thematic analyses.	Acceptance, coping, self-management, integration.	The importance of having a support network is essential for adequate coping, the importance of maintaining social relationships and obtaining emotional and instrumental support.
Arested et al., 2018 [7]	Europe	Family members of patients ill for more than two years, Swedish-speaking. *n* = 11	Qualitative. Narrative interview. Purposive sampling.	Thematic analyses.	Integration.	Need for the family member to be present at meetings with healthcare professionals in order to improve their knowledge of the disease, its management and symptom control.
Kayser et al., 2018 [37]	USA	Quantitative observational studies, colorectal cancer, variables included. *n* = 9 studies. *n* = 808 participants	Systematic review.		Adjustment.	Women in the role showed higher levels of distress. Dyadic approaches are used with the aim of improving communication skills, mutual emotional support and dyadic coping with common cancer-related stresses.
Moral-Fernández et al., 2018 [38]	America, Canada, Asia, Australia	Studies with new primary caregivers, caring for less than 1 year, research using qualitative methodology. *n* = 393 participants	Qualitative metasynthesis.	Thematic analyses.	Acceptance, coping, integration.	Addressing the transition to becoming a family caregiver. Acceptance is necessary to cope.
Whitehead et al., 2018 [39]	Europe, Australia, USA, Asia	Review of studies in English, qualitative or mixed methods. Excluding end-of-life stage. *n* = 19 studies. *n* = 450 participants	Systematic review.		Self-management, integration, adjustment.	Encompasses changes in the context of LTCs’ management from a family perspective.
Ambrosio et al., 2020 [18]	Europe	Review including studies about scales that measure the process of living with the disease. *n* = 13	Integrative review.		Acceptance, coping, self-management, integration, adjustment.	This review highlights a need to further study scales that assess explicit aspects of families living with LTCs
Kusi et al., 2020 [40]	Africa, Asia, USA	Review including quantitative, qualitative and mixed-method studies involving family caregivers of breast cancer patients. N = 19 *n* = 2,330 participants	Systematic review.		Acceptance, coping, self-management.	Addresses the importance of the role of the family caregiver in symptom management. It includes the importance of the economic burden of a sick family member, highlighting the need for policies aimed at considering the family with sick patients. Knowledge as a key aspect in coping with the role of caring.
Gillis et al., 2019 [41]	Europe, USA, Asia	Review of systematic reviews on family participation in the care of patients with chronic diseases. *n* = 10 *n* = 22,242 participants	Reviewing the systematic reviews.		Self-management.	Family caregiver was often included as a substitute for the healthcare provider and the healthcare system. Family members were used as surrogates for professionals to provide care, monitor or encourage the patient to achieve goals.
McGliton et al., 2018 [42]	Europe, USA, Asia, New Zeland	Review including studies in adults >55 years, with at least two chronic conditions and their family caregivers. *n* = 36 *n* = 137 participants	Scoping review.		Self-management.	It sets out five basic needs common to families and patients: the need for information; coordination of services and support; preventive, maintenance and restorative strategies; training for older adults, careers and health professionals to help manage the complex conditions of older adults; and the need for person-centered approaches.
Zhaoyang et al., 2018 [43]	USA	Couples and patients diagnosed with knee osteoarthritis living together. *n* = 132	Quantitative. Observational. Cross sectional. Personal interview. Intentional sample.	Actor-Partner Independence Models	Coping.	Communication as a coping strategy in couples with LTCs. The influence of communicating or suppressing concerns between partners on coping and psychological adjustment to the disease
Meiers et al., 2020 [44]	USA	Family member of patient with chronic condition. *n* = 242	Quantitative. Cross sectional. Convenience sample.	FIES:CI ^1^. Descriptive. CES-D	Integration.	Processes and perceptions of family care and living with LTCs also influence the family as a functional system.
Qin et al., 2019 [45]	Asia	Couples and patients with myocardial infarct. *n* = 111	Quantitative. Observational. Cross sectional.	IPQ-R ^2^. Descriptive.	Coping.	The same problem may be felt differently (e.g., the degree of symptom control perceived differently between patients and family caregivers).
Lynch et al., 2018 [46]	Australia	Adult family caregivers from patients with chronic conditions. *n* = 168	Quantitative. Cross sectional, descriptive. Convenience sample.	Descriptive. Pearlin’s model.	Self-management.	Living with an LTC from family perspective is a complex situation largely influenced by the time spent caring.
Sarris et al., 2020 [11]	Australia	Adult family caregivers from patients with chronic conditions. English-speaking. *n* = 12	Qualitative. Personal interview. Intentional sample.	Thematic analyses.	Adjustment.	It addresses the circumstances that led to the caregiving role, caregiving experience (best and worst) and support needs.
Zhang, 2019 [47]	USA	Studies from family functioning, *n* = 51	Qualitative. Concept analyses.	Rodger’s methods.	Adjustment.	Identifies attributes of family functioning.
Ambrosio et al., 2019 [48]	Europe	Adults, family caregivers from patients with Parkinson’s disease. Spanish-speaking. *n* = 450	Quantitative. Cross sectional, descriptive. Consecutive sampling.	PAIS-SR ^3^. SF-36 ^4^. Descriptive.	Adjustment.	Positive family functioning and increased support can contribute to less perceived caregiver strain and greater perceived well-being.
Ucheddu et al., 2018 [49]	Europe	50 years of age or older and who had a spouse who also participated in the SHARE survey during the same period and not institutionalized.	Quantitative. Cross sectional, retrospective. Convenience sample.	Fixed-effects regression models.	Acceptance.	Most informal carers do not have the option to take on the role of carer; it is a situation that arises.
Helgeson et al., 2019 [50]	Not specified	Review from studies about community coping and adjustment for adults.	Review. Theory Update and Evidence		Coping, self-management.	Describes the process and construction of the theory of coping with chronic illness and the management of chronic illness together with the patient and family caregiver
Berger et al., 2019 [51]	USA	Spousal care partners of people with Parkinson’s disease. *n* = 20	Qualitative. Grounded theory. Pourposeful sample	Thematic analyses. Glaser and Strauss framework.	Acceptance, coping and self-management.	Caring for patients requires caregivers to take care of themselves physically and emotionally. To maintain a proper balance, the inherent social role of the human being needs to be maintained
Riffin et al., 2018 [52]	USA	Family caregivers living with patients of chronic conditions. *n* = 20	Qualitative. Individual interview. Convenience sample.	Thematic analyses.	Self-management.	This study shows the advantages of using a dyadic approach to better understand care relationships.
Faronbi et al., 2019 [53]	Africa	Family caregivers living with seniors with chronic conditions. *n* = 15	Qualitative. Individual interview. Convenience sample.	Thematic analyses.	Self-management.	Family caregivers provide a range of support to their loved ones ranging from help with daily living activities, financial, psychological and spiritual support.
Bertschi et al., 2021 [54]	Europe	Studies of relatives were one of them have LTCs. *n* = 36	Systematic review.		Acceptance, coping, adjustment.	Dyadic coping and communication help to buffer the stress experienced by couples due to chronic deteriorating health.
Gibbons et al., 2019 [55]	USA	Family caregivers and patients with cancer. *n* = 12	Mixed methods. Qualitative and quantitative.	Thematic analyses. FCI scale ^5^, neuro-QoL ^6^, MHC-SF ^7^.	Coping, adjustment.	The diagnosis of LTCs (cancer) affects interpersonal relationships, social networks, finances and functioning of patients and their family caregivers
Roberts and Struckmeyer., 2018 [56]	USA	Patient–caregiver dyads of multiple health conditions. *n* = 20	Part of a mixed-methods study. Qualitative. Individual interview.	Constant comparative method.	Acceptance, adjustment.	Many caregivers report that they derive significant emotional and spiritual rewards from their caregiving role, and others also experience physical and emotional problems directly related to the stress and demands of daily caregiving.

^1^ FIES:CI: Family Integration Experience Scale: Chronic Illness. ^2^ IPQ-R: Revised Illness Perception Questionnaire. ^3^ PAIS-SR: Psychosocial Adjustment to Illness Scale. ^4^ SF-36: Shor form-36. ^5^ FCI Scale: Functional Comorbidity Index. ^6^ Neuro-QoL: Quality of Life in Neurological Disorder. ^7^ MHC-SF: Mental Health Continuum-Short Form.

**Table 5:** Quotes and text from findings: reviewed papers.

Themes and Subthemes	Deductive/ Inductive	Quotes (Q) and Text (T) for Findings. (Reference. Page)
Theme 1: Attributes		
1.1. Acceptance	Deductive	T1. “when an individual has accepted the reality of situation and has ideas about the cause of the problem, accepting the reality of life and all experiences: good or bad” … “is a final stage of adjustment”. ([33], p.3)
		Q1. “Yes, what else can we do? Just accept it submit to a fate like this.” ([33], p.4)
		T2. “Despite describing a situation that was imposed upon them, several of the informants still discussed accepting these changes to daily life. The transition had been successive; they had time to adapt. Even in this exposed situation, some still felt a mutual responsibility. In certain aspects, their relationship had even become strengthened, as helping their loved one and being needed were important to them. Acceptance, although not easy, was a viable coping strategy. Those who emphasized acceptance described how they actively tried to focus on attainable goals rather than letting frustration overwhelm the situation”([35], p.2)
		T3. “The participants both accepted and distanced themselves from the constant challenges of everyday life.” ([36], p.6)
		Q2. “We’ve both gotten to the age, so we’re fully aware that everything might not be like before... it’s well... almost a sort of acceptance” ([33], p. 5)
		T4. “Acceptance is to assume and normalize the role of caregiver, being the final stage of conversion into family caregiver” They mention the new situation that they have to assume and describe the day of onset of their family member’s illness as crucial to their lives and as unpredictable. ([38], p. 5)
		T5. “Given all the problems and needs that family caregivers have, they show different ways of tackling the changes that occur when they begin caring for their relatives. Among these ways that can be adopted of tackling the issue, we find there is generally acceptance of or resistance to the care situation” ([38], p. 5)
		T6. “acceptance of the disability and its consequences also seemed to favor positive dyadic coping. For instance, Smith and Shaw (2017) concluded that PD couples fared well when they assimilated PD into their lives, that is, when couples acknowledged that PD required changes to their lifestyle. This allowed patients to retain more agency and thus provided them with more opportunities to be involved in coping. In contrast, lack of acceptance hindered constructive dyadic coping.” ([54], p. 16)
		T7. “Acceptance is the ability to step back from a caregiving situation to assess the entirety of the situation” ([56], p. 4)
		Q3. “I mean I feel like I’m doing something, trying to do something. There are lots of days when you’re not doing a good job, but at least you’re trying” ([56], p. 8)
1.2. Coping	Deductive	T8. “Coping is dynamic and involves aspects such as the nature of the stressor or the stimulus itself, personal characteristics and external resources such as the support received” ([33], p. 8)
		T9. “Coping is achieved by balancing a sense of purpose in daily activities while maintaining control, even though the family member’s health is constantly changing” ([36], p. 2)
		T10. “to implement strategies to minimize the negative effects of caregiving that allow them to cope with the problems that arise during care” ([38], p. 11)
		T11. “Following trying to normalise the situation of care, caregivers may adopt coping strategies focused on solving problems, which increases safety in the care they provide.” ([38], p. 10)
		Q4. “I think that every time we get a little bit further away, it makes us more secure. It is like dangerous waters and we are gradually sailing out of them.” ([38], p. 10)
		T12. “acceptance of the disability and its consequences also seemed to favor positive dyadic coping” ([54], p. 16)
		T13. “couples fared well when they assimilated PD into their lives, that is, when couples acknowledged that PD required changes to their lifestyle. This allowed patients to retain more agency and thus provided them with more opportunities to be involved in coping.” ([54], p. 16)
		T14. “concepts of self-efficacy, hopefulness, and stress resistance become necessary components of coping capabilities, a complex construct which is often referred to as resilience” ([56], p. 1)
		T15. “Caregiver resilience may be termed as the use of successful coping strategies used by informal and formal caregivers, shifting from the burden perspective to a resilience perspective.” ([56], p. 1)
		T16. “religious coping such as putting one’s faith in God was vital in improving the quality of life among caregivers. Two of the studies further reported that caregivers reported that being religious provided them with meaning in their caregiving roles”. ([40], p. 15)
1.3. Self-Management	Deductive	T17. “importance of support from the formal care systems to help them manage patients’ symptoms in the home setting” ([40], p. 14).
		T18. “Studies in this review highlighted the significant role played by caregivers in symptom management” ([36], p. 15)
		T19. “Family members were used as substitutes for professionals to deliver needed care, monitor, or encourage the patient to obtain goals.” ([41], p. 18)
		T20. “They experienced difficulties managing the care of their family member, but they felt they had no other option. Some caregivers found themselves ensuring their family members kept their appointments, took over medication and nutrition management, and were instrumental in ensuring the person maintained their dignity, particularly at the end of life.” ([42], p. 27)
		T21. “The increase in additional instrumental activities of daily living inherent in the family caregiver role increases the difficulty in finding a balance between participation in social, leisure, and productive activities” ([51], p. 7)
		Q5. “I’m not very good at cutting out time for myself and I have to do it first thing in the morning or my day goes to heck in a hand basket…it’s very empowering to take care of yourself first thing, and then, once you’ve taken care of yourself, then you can take care of all the many disasters that we face every day.” ([51], p. 6)
		T22. “Family caregivers find themselves balancing the resources and demands available to them to manage their daily lives” ([36], p. 6)
		T23. “Previous knowledge on breast cancer aided caregivers to cope effectively in their caring role” ([40], p. 15)
		T24. “Adapting to constant changes and an uncertain future”([36], p. 5)
		T25. “Family support for patients with CD greatly contributes to the management of such a condition” ([32], p. 6). “information and support that would enable the carer and patient to plan for events that might arise in the future ”([32], p. 6)
		T26. “Caring for a family member with Parkinson’s disease keeps the caregiver at home for a long time. This situation changes the caregiver’s course of action, including performing health self-examinations…[caregivers] generally spend their time giving treatment and neglect to check their own health”. ([33], p. 8)
		T27. “family collaboration in self-management has been related to self-management capacity.” ([39], p. 5)
		T28. “caregivers reported a moderate to severe decline in physical health”([40], p. 16). “caregivers usually decreased their working hours or lost paid jobs as a result of the caregiving role” ([40], p. 14)
		T29. “patients and caregivers became anxious as they were uncertain whether they were making the right choices” (37, p. 27). “Taking on this coordinator role was a source of tension between some older adults and their caregivers as they had conflicting ideas about future plans, and how to stay healthy and safe” ([42], p. 27)
		T30. “Involvement of wider family members helped families to maintain a positive approach and to negotiate how to promote self-management” ([39], p. 9)
		T31. “This balancing act was complicated, because it could change from day to day depending on their spouse’s condition”([36], p. 4). “participants had limited time on their own.” ([36], p. 6)
1.4. Integration	Deductive	T32. “in order to maintain the process and balance of family life, roles must be adjusted when the family system encounters challenges” ([32], p. 6)
		T33. “family perspective involves balancing daily chores, maintaining the support of the social network and adapting to constant changes and uncertain future by balancing” ([36], p. 6)
		T34. “Although the participants accepted the spouse’s illness, they were unsure about how to handle their situation. Some distanced themselves from it all, mentioning that they did not think so much about the illness. They tried to live like normal, because this had a value in itself. However, it was not obvious to all participants how they practically handled their circumstances.” ([36], p. 6)
		Q6. “what do I do? I don’t know, I don’t do anything special”. ([36], p. 6)
		T35. “families living with LTCs are required to co-created a context for living their everyday lives, and this is a continuous ongoing process” ([7], p. 3)
		T36. “personal growth and satisfaction that the care task reports to them, and how it helps them value day-to-day things” ([38], p. 9)
		T37. “family created an environment that valued involvement of the family member in everyday activities and normalisation or striving for as normal a lifestyle as possible” ([39], p. 10)
		T38. “processes used by family members to adapt to the realities and uncertainties of chronic illness across the evolving family life cycle in the context of chronic illness.” ([44], p. 3)
		T39. “Families may create positive meaning in the context of the chronic illness experience over time, learn to incorporate illness management into family routines, make adaptations to construct their own subjective illness meanings, and develop strategies for coping with illness management”. ([44], p. 2)
		T40. “[Families] evolve patterns of caring practices [57] to normalize the illness and family life surrounding illness management”. ([44,57], p. 2)
		Q7. “The joy I feel is when I see that she is feeling better, for example, that her old self may show up sometimes. It makes me and her happy. So, you have got to enjoy these little moments.” ([35], p. 3)
		T41. “Still, many informants also depicted moments of happiness. Several authors have identified universal sources of meaning, and such sources are still available, even in the case of severe illness. It was not surprising that, for example, children and friends were mentioned in a positive context, as good relations are a vital source of meaning. However, to gain access to such sources of meaning, the family had to adapt to the new situation. When this was the case, moments of joy were possible.” ([35], p. 4)
1.5. Adjustment	Deductive	T42. “The caregivers’ adaptation to the condition of their family member with Parkinson’s disease is expressed through the acceptance of the condition and an effort to live with the situation.” ([33], p. 3)
		T43. “Psychosocial adjustment to a complex and disabling long-term condition like PD is a complex, dynamic, cyclical and interactive process in which different factors and mechanisms play key roles” ([48], p. 2)
		T44. “Adjustment to a “new normal” requires patients with chronic health conditions to cope with disabling health impairments on a daily basis, for example, by following a treatment regimen, managing the financial impact of treatments, or changing leisure time activities and social interactions to accommodate the impairment” ([54], p. 2)
		T45. “Multiple factors make the adjustment to the caregiving role particularly hard, as the caregiver balances this role with other demands, including child rearing, careers, and relationships” ([56], p. 1)
		Q8. “Some participants had confidence in their ability to handle the situation, and others thought it would work out in the future. As new problems arose, the participants found it natural to identify practical solutions.” ([36], p. 6)
		T46. “high motivation to adhere to his or her caregiving role. In this situation, cultural and religious beliefs and values are considered as incentives for caregivers so that instead of thinking about the difficulties of providing care, caregivers adhere to their cultural and religious beliefs as well as the value of caregiving.” ([34], p. 8)
		T47. “family functioning is defined as family members’ ability to maintain cohesive relationships with one another, fulfill family roles, cope with family problems, adjust to new family routines and procedures, and effectively communicate with each other”. ([47], p. 9)
		T48. “The mere presence of a family caregiver across the cancer trajectory can have a positive impact on the patient’s life and the adoption of healthier habits” ([50], p. 1). “developing interventions at a dyadic level, researchers and medical research partners have the potential to encourage dyadic resilience and sustain partnerships from cancer treatment into survivorship.” ([50], p. 22) “provides a unique opportunity to build on the positive tendencies inherent in dyadic partnerships already engaging in role adjustment and mutuality development in the midst of uncertainty” ([55], p. 2)
Theme 2. Mechanisms		
2.1. Awareness of the situation of change created by an LTC.	Inductive	Q9. “I will serve him (patient) by all means... I have never said I’m tired because he is not only my husband but ‘... also because he is a of wealth and happiness for me and my kids.’ I have a lot of respect for his dignity and status (Wife, 10 years of care).” ([34], p. 4)
		T50. “caregivers experienced a loss of normal life.” ([40], p. 14)
		T51. “How even the most successful suffer tremendous stress due to illness and changing life circumstances” ([55], p. 21)
		T52. “The caregiver must make timely adaptations to adopt the caregiver role within the family system, especially during times of severe exacerbation of the illness.” ([32], p. 6)
		T53. “it is important to recognize that what is important for caregivers‘ health is not only transitioning into caregiving, but also the duration of care. In other words, some caregivers could easily cope with a relative short time of caregiving, but beyond that time it starts to have its negative consequences on individual health.” ([49], p. 8)
		T54. “Participants stated that they experience a disruption in the family process due to the inability to effectively combine the caregiving activities with their family and personal daily demand. The daily routine of care of the elderly impedes on caregivers’ attendance to their own daily business, family matters and other personal obligations.” ([53], p. 3) “Caregivers in this study spend substantial numbers of hours in providing care daily for their sick elderly. They assist in activities of daily living such as feeding, grooming, changing of position, medication and running errands. Almost one-third of the respondents in this study claimed that they spend almost the entire day caring for the sick elderly” ([53], p. 5)
		T55. “A certain concern or burden is evident due to the constant apprehension about the present and future of their affected family member” ([32], p. 6)
		T56. “in different health conditions, partners often feel overwhelmed by their new “identity” as caregivers and with caregiving tasks”. ([54], p. 17)
		Q10. “becoming a caregiver is not a normatively expected transition and, therefore, is not preceded by systematic preparation” ([49], p. 2)
2.2. Personal networks	Inductive	T57. “In fact, a positive family functioning and a greater support between family members may contribute to a lower perception of burden in caregivers and to a higher perception of well-being.” ([18], p. 1)
		T58.“information and support that would enable the carer and patient to plan for events that might arise in the future” ([32], p. 6)
		T59.“collaboration was necessary for the family to feel secure in their ability to handle life with illness in the best way possible. When the families felt they could collaborate in the caring process, it contributed to feelings of confidence, and that their input could influence and contribute in the situation” ([7], p. 12)
		T60. “family collaboration in self-management has been related to self-management capacity.” ([39], p. 5)
		T61.“ Individuals in stressful situations such as caregiving can benefit from social support networks as they can provide the resources that help them manage their situation” ([56], p. 4)
		Q11. “The caregiver group is a godsend, because sometimes you’ve just got to dump and you can do it there. It makes me feel better because I know I’m not alone. Every other one of those wives is going through what I’m going through, it’s the neatest, tiredest looking group of women I’ve seen. We have days when we laugh and cry, it’s like this little amount of light. Without the groups, I wouldn’t have made it.” ([56], p. 7)
		Q12. “It does feels really good to just get a break, I just feel guilty, I’m not gonna lie.” ([56], p. 7)
		T62. “The study respondents were unanimous in reporting that their feelings of isolation were amplified with the increase in their caregiving responsibilities. “([56], p. 5)
2.3. Information and education about the LTCs.	Inductive	T63. “Need for information about the illness, care and prognosis of the family member, and the training to develop the necessary skills to perform the task of caring in the best possible way, is reflected in the requests of family caregivers to health personnel” ([38], p. 9)
		T64. “Family carers in the present study assumed the provision of care with no knowledge or experience in dealing with the disease, the decision-making, the management of the complications and the interpersonal challenges yet to come, all of which could be facilitated if early-stage information was available regarding the progression of the disease, information and support that would enable the carer and patient to plan for events that might arise in the future” ([32], p. 5)
		T65. “The provision of informational support aided in decreasing caregiving burden among the caregivers.” ([40], p. 17)
2.4. Personal conditions	Inductive	T66. “The results showed that most participants have reliable coping mechanisms such as accepting their parents’ conditions and relating the event to spiritual aspects.” ([33], p. 7)
		T67. “religious coping such as putting one’s faith in God was vital in improving the quality of life among caregivers. Two of the studies further reported that caregivers reported that being religious provided them with meaning in their caregiving roles”... “Previous knowledge on breast cancer aided caregivers to cope effectively in their caring role” ([40], p. 15)
		T68. “The caregiver... must have a high motivation to adhere to his or her caregiving role. In this situation, cultural and religious beliefs and values are considered as incentives for caregivers so that instead of thinking about the difficulties of providing care, caregivers adhere to their cultural and religious beliefs as well as the value of caregiving”. ([34], p. 4)
		T69. “Their beliefs that caring for an older adult is an investment serve as a motivation to continue despite all odds”. ([53], p. 7)
		T70. “Financial challenges such as lack of transportation, loss of a paid job, and high treatment cost were also fundamental sources of stress for caregivers” ([40], p. 15)
		T71. “Families who view the providing of care for the elderly as an obligation experienced pride and increased satisfaction, and expressed a positive response.” ([33], p. 8)
2.5. Attitude to life	Inductive	T72. “Involvement of wider family members helped families to maintain a positive approach and to negotiate how to promote self-management” ([39], p. 9)
		T73. Earlier experiences of success in life or of being optimistic served as resources that the participants used to manage daily life. ([36], p. 5). Q 13. “I’m a pretty positive person…I don’t let the situation get the better of me.” ([36], p. 5)
		T74. “They seek to reduce the negative effects by trying to take care of strategies to mantain emotional positivity… being positive and optimistic… mantain spiritual support” ([38], p. 10)
		T75. “[Acceptance] It is characterised by a positive attitude, recognition or appreciation of individual values and acknowledgement of one’s own behaviour” ([33], p. 7)
2.6. Communication.	Inductive	T76. “Changes in openness were described within some of the families and communication patterns were altered”. ([35], p. 2)
		T77. “Family functioning influences family member health, and discrepant perceptions of family functioning contribute to poor psychological health.” ([44], p. 2)
		T78. “However, communication was also described as crucial to the relationship” ([36], p. 7)
		T79. “Those at risk for relationship distress could be taught skills that will help them cope, including helpful communication skills such as validation, as this was found to be associated with reduced distress” ([37], p. 14)
		T80. “Family Health Conversations (FHC) are an appropriate way to involve families and attain a family centered care, let them tell their story, and enhance family well-being...It can also make it easier for families to handle challenges faced due to illness, and therefore, it also contributes to overall family well-being” ([7], p. 27)
		Q13. “We try to talk more, not less. For you need to talk about things to lighten them up, otherwise. Because there’s always something to take care of.” ([36], p. 5)
